# The burden of transport injury and risk factors in India from 1990 to 2019: evidence from the global burden of disease study

**DOI:** 10.1186/s13690-022-00962-8

**Published:** 2022-09-05

**Authors:** Deepak Kumar Behera, Sanjay Kumar Singh, Dinesh Kumar Choudhury

**Affiliations:** 1 Department of Economics, Birla School of Social Sciences and Humanities, Birla Global University, Bhubaneswar, Odisha 751029 India; 2 Indian Institute of Management Lucknow, Lucknow, 226013 India; 3 Department of Economics, Sri Sathya Sai Institute of Higher Learning, Anantapur, 515134 India

**Keywords:** Transport injury, Burden of disease, Traffic accident, traffic rules, India

## Abstract

**Background:**

India is one of the fastest-growing developing economies associated with many socio-demographic challenges that include a high density of population, growing urbanization, and poor road infrastructure. These challenges might lead to the cause of injury, especially transport related. Therefore, we aim to analyze the burden of Transport Injury (TI) and associated risk factors in India using the required data from 1990 to 2019.

**Methods:**

This study has used the latest Global Burden of Disease Study (GBD) 2019 data set and estimated TI-related incidence rate, mortality (death) rate, and Disability-Adjusted Life Years (DALYs) lost for India over the period from 1990 to 2019. The latest round of GBD survey-2019 provides information about 369 diseases and injuries and 87 risk factors across age groups and gender.

**Results:**

Around 25% of the death rate of all ages was caused due to TI in 2019, significantly higher than in 1990 (20%). However, between 1990 and 2019, the DALYs rate per 100,000 people due to TI decreased slightly by 1.6% for all ages and both gender while more reduction has been observed in under 5- and 5–14-years age groups. On the contrary, the incidence rate and DALYs rate had increased substantially in the age group above 50 years which could be a serious issue for the safety of aging people. By analyzing the sub-cause of TI, we found that motorcyclist road injuries and pedestrian road injuries have been major causes of deaths in India during the last three decades. Further, we have found four risk factors associated with environmental change, occupational hazard, behavioral risk, and metabolic risk that cause TI injuries.

**Conclusions:**

TI-related disease burden has not been reduced over the years in India despite improvements in road infrastructure and digital technology. Improvement in transport policies; awareness about traffic rules and laws among citizens, and improvement in governance in the road & transport sector could change the behavioral risk factors of TI and reduce population unwanted death and suffering.

## Background

As per the latest Global Burden of Diseases – 2019 (GBD) study, road injury was having the eighth position among the top twenty leading causes of disease burden in 1990 and now it is at the seventh position in 2019 in the world [1]. The burden of injury exhibits a similar trend in India as well [2]. Past studies argue that an increase in deaths and incidence cases due to road accidents is going to be a serious public health issue in India [3, 4]. This is because India is known as a poor public health spender and disease-related spending has never been a policy agenda of the government [5]. Apart from the low disease-specific health expenditure, other socio-economic factors such as rising urban population, poor quality roads, and increasing crime rate aggravate the burden [6, 7]. Additionally, as India progresses in terms of economic prosperity, the behavioral factors have also changed similarly leading to more consumption of alcohol, drugs, and smoking. Eventually, that was considered a risk factor for road injury by earlier studies [7, 8]. Not only behavioral factors, but a few institutional factors such as poor roadways, and weak enforcement of traffic policies were also the major factors of road accidental injury [9, 10]. Apart from the behavioral and institutional factors, there are a few factors such as temperature, occupational injury, and metabolic factors that also play a role in the cause of road accidental injury.

As per our understanding of the literature, we have found that no study has explored all types of risk factors including environmental risk, occupation risk, behavioral risk, and metabolic risk associated incidence cases. Additionally, earlier studies only focus on road accident injury-related death and incidence cases in India, but no one has explored the burden of disease indicators – Disability Adjust Life Years (DALYs) because the burden of disease parameters only determines what could be the level of demand and supply of health care. Therefore, this study analyses the burden of disease, and associated risk factors of Transport Injury (hereafter TI). The study has estimated incidence rate, mortality rate, and DALYs rate across age groups and gender-wise over the period from 1990 to 2019 using the latest GBD-2019 data. Additionally, the study has estimated the sub-cause of TI-related mortality, incidence, and DALYs. All estimation has used the disaggregated data by the type of road user, gender, and age to quantify the burden of injuries in terms of mortality and disability-adjusted life years (DALYs).

## Methods

The study has used the latest round of GBD study 2019, which is a comprehensive database related to communicable diseases, non-communicable diseases, and injuries over the period from 1990 to 2019 across the 204 country samples [11]. The latest round of GBD survey-2019 provides information about 369 diseases and injuries and 87 risk factors across age groups and gender-wise. In this study, we have adopted the data related to TI, which is a sub-category of injuries, and GBD 2019 generated a complete set of estimates for TI-specific mortality rate, DALYs, and incidence rates across age groups, and gender in India.

**Fig. 1:**
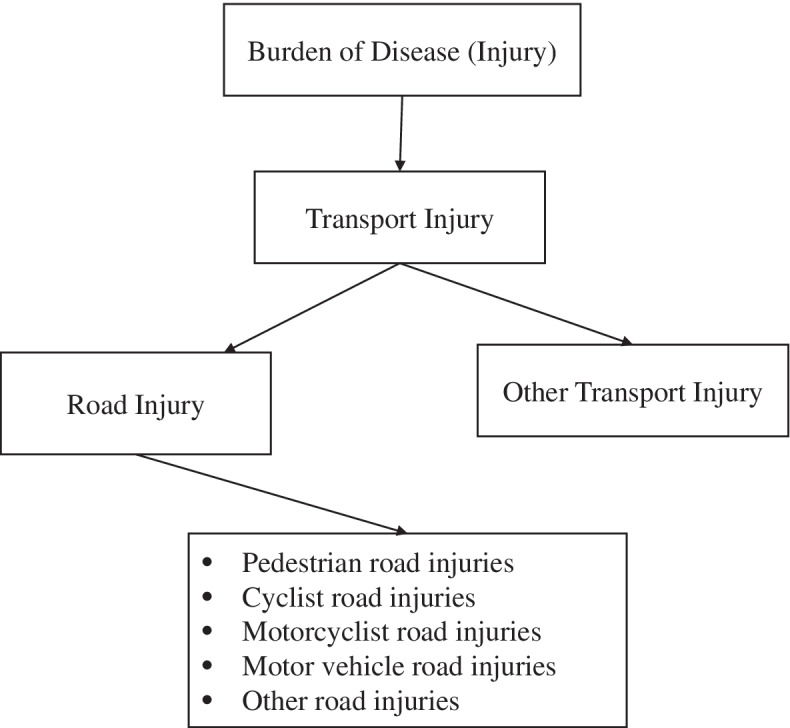
Cause hierarchy of transport injuries in GBD 2019

For this study, we have downloaded GBD 2019 estimates for India from the publicly available online database on the Global Health Data Exchange (GHDx) and GBD results tools from the Institute of Health Metrics Evaluation [11]. To describe the data, we have reviewed a list of GBD cause hierarchies of TI. We used the GBD 2019 level 3 causes of disease related to the transport sector and sub-causes of TI which is level 4 causes of disease. TI estimates included six sub-cause of injuries that includes Pedestrian Road injuries, Cyclist Road injuries, Motorcyclist Road injuries, Motor vehicle road injuries, other road injuries, and other transport injuries. The analysis has been done across the period, across age groups, and gender in India. Figure 1 shows the cause hierarchy of TI. Additionally, we have estimated the incidence rate, mortality rate, and DALYs using a certain formula. Table 1 shows the indicators and measurement criteria used in the study.

**Table 1:** Definition of variables and measurement

Measure	Number	Percent	Rate
Deaths (mortality)	Number of deaths in the population	The proportion of deaths for a particular cause relative to deaths from all causes	Deaths per 100,000 population
Disability-adjusted life years (DALYs)	Number of DALYs in the population	The proportion of DALYs for a particular cause relative to DALYs for all causes	DALYs per 100,000 population
Incidence	Number of new cases in the population	The proportion of new cases of a particular cause relative to cases from all causes	New cases per 100,000 population

Source: Author’s estimation from GBD -2019 (IHME, 2020)

## Results

**Fig. 2:**
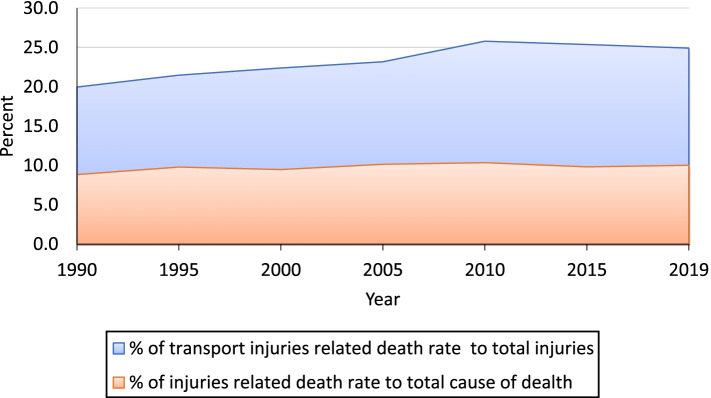
Time-series trends of transport injuries related to total death (mortality) rate in India of all ages

Our results section is divided into three measurements of TI that include incidence rate, the death rate (mortality rate), and DALYs rate of all age groups and across gender from the period from 1990 to 2019. Figure 2 shows the time series trends of transport injuries and the overall injury total death rate in India. It shows that the percentage share of TI death to total Injury death has increased from 20% in 1990 to 25% in 2019. Similarly, the percentage share of injuries death to total cause of death has increased slightly from 8.8% in 1990 to 10.0% in 2019. Figure 2 shows the share of Transport Injury to total injury-related deaths in India; it reveals the increasing trend from 1990 to 2019. The share of injury to the total cause of diseases has also increased over the same period. It can easily be inferred that transport sector-related injury would become a serious burden for India if the country does not resolve the associated risk factor of the accident.

### Incidence cases of transport injury and its sub-cause

Table 2 shows that the total incidence rate of TI was 1947 [CI:1600–2345] in 1990 and it has increased to 2681 [CI: 2194–3234] in 2019, an increment of 38% in three decades. This table also reveals shows that there is a reduction in the incidence rate from 1990 to 2019 in the age group under 5 and 5–14 years, though the reduction is significantly more for females than males. On the contrary, there is a substantial increase in incidence rate from 1990 to 2019 in the age group 15–49, 50–69, and 70+ years, though the increase is lower for females than males. Across gender, the female incidence rate decreased by around 15.8% in the age group under 5 and 13.7% in the age group 5–14 years. But incidence rate of TI increased in the working-age group as well as the aging population in 2019 across both genders in India.

**Table 2:** Incident, death, and DALYs rate for transport injuries in India for each age group and gender

Age group	Gender	Incident rate (per 100,000)		Death rate (per 100,000)		DALYs rate (per 100,000)	
		1990	2019	1990	2019	1990	2019
All Ages	Male	2520 (2051–3052)	3610 (2918–4387)	25 (20–28)	26 (17–31)	1719 (1449–1922)	1787 (1360–2119)
All Ages	Female	1325 (1094–1595)	1704 (1416–2037)	9 (8–10)	8 (6–9)	703 (613–797)	608 (504–715)
All Ages	Person	1947 (1600–2345)	2681 (2194–3234)	17 (14–19)	17 (13–20)	1232 (1070–1374)	1212 (978–1419)
Under 5	Male	443 (323–602)	441 (314–605)	11 (7–14)	3 (2–4)	955 (647–1229)	237 (169–354)
Under 5	Female	297 (197–434)	250 (169–368)	9 (7–12)	3 (2–4)	800 (611–1028)	240 (176–353)
Under 5	Person	373 (266–513)	350 (252–483)	10 (8–13)	3 (2–4)	881 (658–1100)	238 (180–344)
5–14 years	Male	1182 (823–1718)	1156 (768–1712)	8 (5–9)	4 (2–5)	642 (473–791)	321 (228–404)
5–14 years	Female	850 (594–1240)	734 (501–1089)	5 (4–6)	2 (2–3)	422 (328–530)	195 (153–240)
5–14 years	Person	1023 (714–1490)	955 (643–1398)	6 (5–8)	3 (2–4)	537 (414–655)	261 (206–320)
15–49 years	Male	3753 (2868–4752)	4953 (3709–6250)	31 (26–35)	30 (21–37)	2271 (1971–2527)	2241 (1683–2639)
15–49 years	Female	1773 (1325–2236)	2083 (1572–2622)	8 (7–9)	6 (4–7)	687 (595–783)	547 (450–642)
15–49 years	Person	2802 (2129–3544)	3561 (2673–4493)	20 (17–22)	18 (13–22)	1510 (1333–1682)	1419 (1123–1650)
50–69 years	Male	2729 (1952–3582)	3802 (2792–5032)	47 (37–54)	43 (28–55)	2583 (2157–3011)	2733 (2114–3362)
50–69 years	Female	1796 (1290–2373)	2501 (1803–3306)	18 (15–21)	18 (14–22)	1221 (1010–1442)	1322 (1071–1591)
50–69 years	Person	2289 (1639–3021)	3148 (2259–4153)	34 (28–38)	30 (22–37)	1940 (1651–2253)	2024 (1627–2455)
70+ years	Male	2256 (1561–3156)	2957 (2058–4034)	72 (54–82)	64 (42–78)	2388 (1947–2838)	2626 (2080–3224)
70+ years	Female	993 (676–1420)	1104 (752–1534)	34 (26–41)	31 (25–37)	1265 (1039–1500)	1362 (1098–1656)
70+ years	Person	1617 (1112–2274)	1971 (1363–2724)	53 (44–59)	46 (36–54)	1820 (1516–2142)	1954 (1566–2395)

*Note*: Parenthesis denotes a 95% uncertainty interval of lower and upper limits

Table 3 shows all ages incidence per 100,000 people for males and females, and both put together for transport injury sub-causes in the Global Burden of Disease Study (GBD) cause hierarchy between 1990 and 2019. It shows that cyclist road injuries were a major cause of road accidents in 1990 and still it has a major share in transport injuries in 2019 for males as well as females. Moreover, the second cause of TI is motorcyclist road injuries followed by pedestrian road injuries. Table 3 shows that the total motorcyclist road injuries incidence rate has increased from 300 [CI: 233–387] in 1990 to 548 [CI: 428–697] in 2019. Similarly, the incidence rate of Pedestrian road injuries has increased from 380 [CI: 286–495] in 1990 to 519 [CI: 397–661] in 2019.

**Table 3:** Incident, death, and DALYs rate for the components of transport injuries in India for all ages and gender

		Incident rate (per 100,000)		Death rate (per 100,000)		DALYs rate (per 100,000)	
Types of Transport injury	Gender	1990	2019	1990	2019	1990	2019
Cyclist road injuries	Male	1184 (921–1503)	1529 (1184–1941)	1.7 (1.2–2.4)	1.7 (1.1–2.2)	211 (162–271)	239 (182–303)
	Female	l497 (377–651)	511 (392–671)	0.2 (0.1–0.3)	0.2 (0.2–0.3)	64 (48–82)	68 (51–88)
	Person	854 (661–1096)	1033 (799–1317)	1.0 (0.7–1.4)	1.0 (0.7–1.2)	140 (110–178)	156 (118–198)
Motor vehicle road injuries	Male	387 (277–498)	582 (422–764)	6.1 (4.8–8.0)	5.8 (4.2–7.5)	388 (314–485)	371 (296–457)
	Female	241 (177–314)	375 (277–486)	2.1 (1.5–2.9)	2.2 (1.7–2.8)	144 (109–185)	147 (120–179)
	Person	317 (231–406)	481 (352–630)	4.2 (3.5–5.2)	4.0 (3.3–4.9)	271 (232–326)	262 (221–313)
Motorcyclist road injuries	Male	396 (305–510)	755 (585–970)	5.8 (4.4–7.1)	7.8 (4.8–9.8)	420 (344–493)	581 (428–703)
	Female	195 (150–253)	330 (260–418)	1.1 (0.7–1.4)	1.5 (0.9–1.9)	109 (83–133)	151 (119–186)
	Person	300 (233–387)	548 (428–697)	3.5 (2.8–4.2)	4.7 (3.0–5.8)	271 (226–317)	372 (283–445)
Pedestrian road injuries	Male	436 (324–568)	615 (462–790)	8.3 (5.5–10.4)	7.7 (4.8–9.9)	518 (370–636)	443 (306–544)
	Female	320 (244–409)	418 (323–526)	4.3 (3.5–5.3)	3.0 (2.4–3.6)	318 (259–383)	198 (165–233)
	Person	380 (286–495)	519 (397–661)	6.4 (4.8–7.7)	5.4 (3.9–6.6)	422 (333–502)	324 (254–384)
Other road injuries	Male	36 (24–52)	54 (35–78)	0.2 (0.2–0.3)	0.2 (0.1–0.2)	14 (10–19)	11 (8–13)
	Female	37 (24–56)	45 (28–67)	0.1 (0.0–0.1)	0.1 (0.0–0.10	7 (4–8)	6 (5–8)
	Person	37 (24–53)	50 (32–72)	0.1 (0.1–0.2)	0.1 (0.1–0.1)	10 (8–13)	8 (7–10)
Other transport injuries	Male	82 (64–104)	75 (59–94)	2.8 (1.9–3.3)	2.6 (1.6–3.2)	168 (122–199)	142 (93–172)
	Female	35 (26–46)	320 (244–409)	0.9 (0.7–1.1)	0.7 (0.6–0.9)	62 (46–74)	38 (32–44)
	Person	59 (46–77)	50 (40–64)	1.9 (1.5–2.2)	1.7 (1.1–2.0)	117 (93–134)	91 (66–107)

*Note*: Parenthesis denotes a 95% uncertainty interval of lower and upper limits

### Deaths of transport injury and its sub-cause

Table 2 shows that the total mortality (death) rate of TI was 17.1 [CI:14–19] in 1990 which got reduced slightly to 16.9 [CI:13–20] in 2019. It shows that there was a substantial reduction in mortality between 1990 and 2019 in the age group under 5 and 5–14 years. On the contrary, there was a less percentage of reduction in mortality between 1990 and 2019 in the age group 15–49, 50–69, and 70+ years. But there is a gender-wise difference in the reduction of mortality rate across age groups. But overall, there is a reduction in the TI-related death rate in India across age groups and gender.

Table 3 shows all ages mortality per 100,000 people for males, females, and total mortality put together for transport injury sub-causes in the Global Burden of Disease Study (GBD) cause hierarchy between 1990 and 2019. It shows that the major cause of mortality is due to motorcyclist road injuries, pedestrian road injuries, and motor vehicle road injuries in 2019. The total person mortality rate of motorcyclist road injuries was 3.5 [CI: 2.8–4.2] in 1990 and increased to 4.7 [CI: 3.0–5.8]. On the other hand, the total person mortality rate of pedestrian road injuries was 6.4 [CI: 4.8–7.7] in 1990 and reduced to 5.4 [CI: 3.9–6.6].

Gender-wise comparison of TI injuries shows that male person injuries are more as compared to female persons in 2019 in all categories of TI in India. We have found that pedestrian road injuries were around 4.3 [CI: 3.5–5.3] in 1990 in the female category which got reduced to 3.0 [CI: 2.4–3.6] in 2019. But in the male category, motorcyclist road injuries were 5.8 [CI: 4.4–7.1] and it got increased to 7.8 [CI: 4.8–9.8] in 2019. Our study finds that motorcyclist road injuries have increased over the years across genders, while cyclist road injuries, other road injuries, and other transport injuries mortality rate has reduced.

### DALYs of transport injury and its sub-cause

Table 2 shows that the total DALYs rate was 1232 [CI: 1070–1374] in 1990 which got reduced to 1212 [CI: 978–1419] in 2019. However, the DALYs rate increased in the male category between 1990 to 2019 though the rate got reduced for the female category. It has been seen that the DALYs rate has increased across gender categories in the age groups 15–49, 50–59, and 70+ while it has substantially reduced in the age group under 5 and 5–14 years.

Table 3 shows all ages DALYs per 100,000 people for male, female, and total persons for transport injury sub-causes between 1990 and 2019. In 1990, motorcyclist road injuries were 271 [CI: 226–317] per 100,000 people and it has increased over the period to 372 [CI: 283–445] per 100,000 people in 2019. But there is a gender-wise difference in the cause of DALYs due to TI in India. Motorcyclist injuries DALYs rate is higher for males than females while pedestrian road injuries are higher for females than males.

### Risk factors of transport injuries

Table 4 shows the Risk factor of TI. As per GBD 2019, there are mainly four risk factors associated with environmental change, occupational hazard, behavioral risk, and metabolic risk that cause TI injuries. We have estimated the risk and cause-specific death rate, and DALYs of all ages and both genders in 2019. The result shows that DALYs and Death rates in 2019 due to high temperature are 33.1 [CI: 11.7–65.2] and 0.7 [CI: 0.3–1.4], respectively. Similarly, DALYs and Death rates due to occupational injuries are 182.7 [CI:132.9–245.1] and 2.4 [CI:1.7–3.4] respectively. Behavioral risk factors due to consumption of alcohol, and smoking are responsible for high death rates and DALYs in India. As per the GBD study, metabolic risk factors include high fasting plasma glucose, high LDL cholesterol, high systolic blood pressure, high body-mass index, low bone mineral density, and kidney dysfunction. In this study, we have found that only one risk factor i.e. low bone mineral density causes TI-related deaths and DALYs in India. Overall, our study finds that all risk factors contribute to 4.7 [CI: 3.5–6.0] and 320.3 [CI: 252.3–402.1] of death and DALYs respectively in India. Additionally, the study has identified major contributing risk factors of TI in India that includes occupational injuries, alcohol consumption, and low bone mineral density.

**Table 4:** Risk factor of transport injuries related DALYs and death rate (per 100,000 population) of all ages and total persons in 2019

Sl. No.	Risk factor	Death	DALYs
1	All risk factors	4.7 (3.5–6.0)	320.3 (252.3–402.1)
A.	Environmental/occupational risks	2.6 (1.9–3.7)	191.6 (139.9–259.1)
A.1	Non-optimal temperature	0.2 (−0.1–0.9)	10.7 (−3.7–42.6)
A.1.1	High temperature	0.7 (0.3–1.4)	33.1 (11.7–65.2)
A.1.2	Low temperature	−0.5 (− 0.9 - -0.1)	−23.5 (−40.9 - -6.5)
B.	Occupational risks	2.4 (1.7–3.4)	182.7 (132.9–245.1)
B.1	Occupational injuries	2.4 (1.7–3.4)	182.7 (132.9–245.1)
C.	Behavioral risks	0.9 (0.5–1.5)	62.9 (35.9–104.1)
C.1	Tobacco	0.1 (0.1–0.2)	6.3 (4.2–8.9)
C.1.1.	Smoking	0.1 (0.1–0.2)	6.3 (4.2–8.9)
C.2	Alcohol use	0.8 (0.4–1.4)	56.9 (29.6–97.2)
D.	Metabolic risks	1.6 (1.2–1.9)	95.5 (74.1–116.0)
D.1	Low bone mineral density	1.6 (1.2–1.9)	95.5 (74.1–116.0)

*Note*: Parenthesis denotes a 95% uncertainty interval of lower and upper limits

## Discussion

This is the first kind of comprehensive study on the burden of transport injuries in India from 1990 to 2019. Since 1990, total injury mortality rates to the total cause of death have declined in India, but the transport injury incidence cases and the mortality rate has increased over time. The magnitude of transport injury burden varies across time by sub-cause, gender, and age group. Our study revealed that the working-age group (age 15–49 years) and old age people (age above 50+ years) experience a higher burden of transport injury cases, mortality, and DALYs over the period and it has substantially increased from 1990 to 2019 in comparison to the age group under 5 and 5–14 years. The concentration of injury burden on the working-age group is a serious public health issue because it would generate an unhealthier workforce in the economy thereby labor productivity and economic growth will deteriorate [12].

Several sub-causes affect the transport injury. Our result shows that cyclist road injury incidence cases increased sharply from 1995 to 2010, then it started declining but is still a major cause of transport injury in 2019 followed by pedestrian and motorcyclist road injuries. On the contrary, the pedestrian road injury death rate is higher over the period followed by motorcyclist and motor vehicle road injuries in India. It shows that road injuries that happened due to pedestrians and motor vehicles are more severe than other cyclist road injuries. Similarly, DALY’s rate is also higher over the period due to pedestrian road injuries followed by motorcyclist and motor vehicle injuries in India. Our finding is like other studies that found that pedestrian and motorcyclist injuries were the main cause of TI injuries and that could be due to poor quality of the road, lack of pedestrian walkways, and lack of road safety awareness in India [13, 14]. Further, few have argued about the behavior of motorcyclists such as not wearing helmets, alcohol consumption, and not obeying traffic signals which are the major factors for motorcycle road injury [15, 16]. Similarly, few have argued some risk factors such as overspeeding, overloading, wrong-side driving, commercial place, and faulty road design could be possible risk factor for road accidents in Indian states [17]. The literature identified a few structural issues that cause speed-related road accidents in India such as drivers without a valid driving license, single-lane roads, uncontrolled junction roads, daylight, and the presence of central divides [18].

From the above results, we can draw a few inferences that there is a huge traffic pressure on-road transportation that leads to a road accidents. This could be due to poor road infrastructure associated with the increasing urban population over the period. Additionally, the standard of road communication has deteriorated that should be improved to save the people. Along similar lines, Stevenson et al. (2020) argue that increasing urbanization and labor mobility demands a safe and clean transportation facility to deal with road traffic injury death in a low-and middle-income country. Table 4 shows a few risk factors for TI-related DALYs and the Death rate in India. Especially, factors due to higher alcohol consumption, occupation injuries, and low mineral density have led to the cause of TI in India. In the corollary of our findings, the past studies also found distributional variation in TI-related death among age groups and gender (Singh, 2020; Dandona, 2020; Gururaj, 2008; Nguyen et al., 2020). Similarly, we have found that alcohol, drug use, and not obeying traffic rules are possible risk factors for TI (Das et al., 2012; Uthkarsh et al., 2012). Few have argued that behavioral change, roadway infrastructure, and enforcement of traffic policies were the main concern among the general road traveler and improvement of these issues would be effective to reduce road injury-related fatality (Jacoby et al., 2016 Gupta and Bandyopadhyay, 2020).

Earlier studies mostly analyze the behavioral risk factors for the cause of road injuries such as consumption of smoking and alcohol (Nguyen et al., 2020; Gupta and Bandyopadhyay, 2020; Uthkarsh et al., 2012) but no studies have estimated or even explored the environmental factors – high and low temperature, occupational injuries, and metabolic factor – low bone mineral density. Similarly, most studies have analyzed road injuries case and death using survey data, but our study is based on injuries due to transportation and associated mortality, DALYs, and risk factors. However, our study has not been able to estimate the effects of socio-economic infrastructure on the occurrences of injury in India due to a lack of data availability which is a limitation of this study.

Although this study has not used other socio-economic factors to examine the possible effects of TI-related deaths, the study would provide a clear snapshot of trends and patterns of injury due to the transportation sector in India. It will be a useful study for both public health researchers as well as urban planners to make a holistic measure to mitigate the future demand and supply of road communication and associated public health issues.

## Conclusions

The study aims to analyze the burden of TI and associated risk factors in India using the required data from 1999 to 2019. It is found that the incidence rate and DALYs rate increased substantially in the age group above 50 years which could be a serious issue for the safety of aging people. It concludes that TI-related new cases, death rate, and the burden have not been reduced over the period in India despite population growth, urbanization, and safe traffic rules in India. It is one apprehension that if road traffic injury would not minimize the populous country like India, it would increase the medical costs and health-related expenses thereby it can push the injured family into poverty.

Therefore, there is a need for a strong policy framework to tackle the issue of transport injuries and related deaths & suffering in India at the regional level. In a similar vision, the government of India has recently amended the Motor Vehicle Act Amendment Bill of 2019 in parliament. This bill is a bold step in the right direction to reduce road crash deaths and ensure the safety of all road users by imposing monetary penalties and punishment on traffic rule breakers. However, a few structural problems might arise in the implementation of road safety rules and regulations in India in terms of awareness and changing the behavior of the citizens. Therefore, the focus should be on the effective implementation of traffic rules on both rural and urban roads by maintaining governance in the transport sector which could reduce transport injuries even if rapid urbanization and population growth.

## Data Availability

Data is available in the public domain for research purposes and not for commercial use. Data can be obtained from the open access repository of the Institute of Health Metrics Evaluation (IHME).
